# Network traits driving knowledge evolution in open collaboration systems

**DOI:** 10.1371/journal.pone.0291097

**Published:** 2023-11-14

**Authors:** Ruqin Ren, Jia He

**Affiliations:** Institute of Cultural and Creative Industry, Shanghai Jiao Tong University, Shanghai, China; The University of Arizona, UNITED STATES

## Abstract

Network interpretation illuminates our understanding of the dynamic nature of cultural evolution. Guided by cultural evolution theory, this article explores how people collectively develop knowledge through knowledge collaboration network traits. Using network data from 910 artifacts (the WikiProject Aquarium Fishes articles) over 163 weeks, two studies were designed to understand how collaboration network traits drive population and artifact-level knowledge evolution. The first study examines the selection pressure imposed by10 network traits (against 11 content traits) on population-level evolutionary outcomes. While network traits are vital in identifying natural selection pressure, intriguingly, no significant difference was found between network traits and content traits, challenging a recent theory on network-driven evolution. The second study utilizes time series analysis to reveal that three network traits (embeddedness, connectivity, and redundancy) at a prior time predict future artifact development trajectory. This implies that people collectively explore various positions in a potential solution space, suggesting content exploration as a possible explanation of knowledge evolution. In summary, understanding the interplay between network traits and content exploration provides valuable insights into the mechanisms driving knowledge evolution and offers new avenues for future research.

## 1. Introduction

Since the earlier work of linguists who constructed evolutionary trees of language development trajectories [1], scholars have seen human knowledge development as a non-random and cumulative evolutionary process that is highly analogous to the way in which biological species evolve [2]. The intricacy and beauty of human culture is explained as a result of how initial beliefs and ideas are mutated, transmitted, and selectively retained over time [2]. This insight is even more intriguing today because of the new forms of information creation and transmission made possible by digital technologies, such as self-organized knowledge collaboration systems [3–5].

A fundamental inquiry in cultural evolution is about *wha*t is driving evolution, because the task of identifying a unit of cultural trait under selection is more difficult than in biological realm [6, 7]. Traits, characteristics, or properties of individuals are often used in biology to define *species* or *types*, but social scientists face high ambiguities in defining cultural traits that drive evolution. A recent discovery in this stream of literature is Hilbert et al. [7], which suggested that researchers should not only examine the content of knowledge and information that is evolving, but also treat the structure of social networks that facilitated cultural adaptations as another important driver. Essentially, to address what drives knowledge evolution, we need to examine both the content of the knowledge created but also the network structure associated with the knowledge under development.

Different fields paid attention to network structural patterns from different theoretical lens. Research at the population level, often conducted by cultural evolution theorists, has identified network traits as a key driver of cultural adaptation outcomes [7]. Price equation was a commonly used tool to quantify the extent of population-level change attributable to certain traits. Research at the individual artefact level, a common focus of information system scholars, has connected structural patterns of social networks producing these artefacts as predictors of content development quantity and quality [5, 8]. However, no research yet has systematically connected the drivers of cultural evolution (population level) directly to the predicted patterns of specific knowledge artifacts’ content development trajectory (individual level).

**Fig 1:**
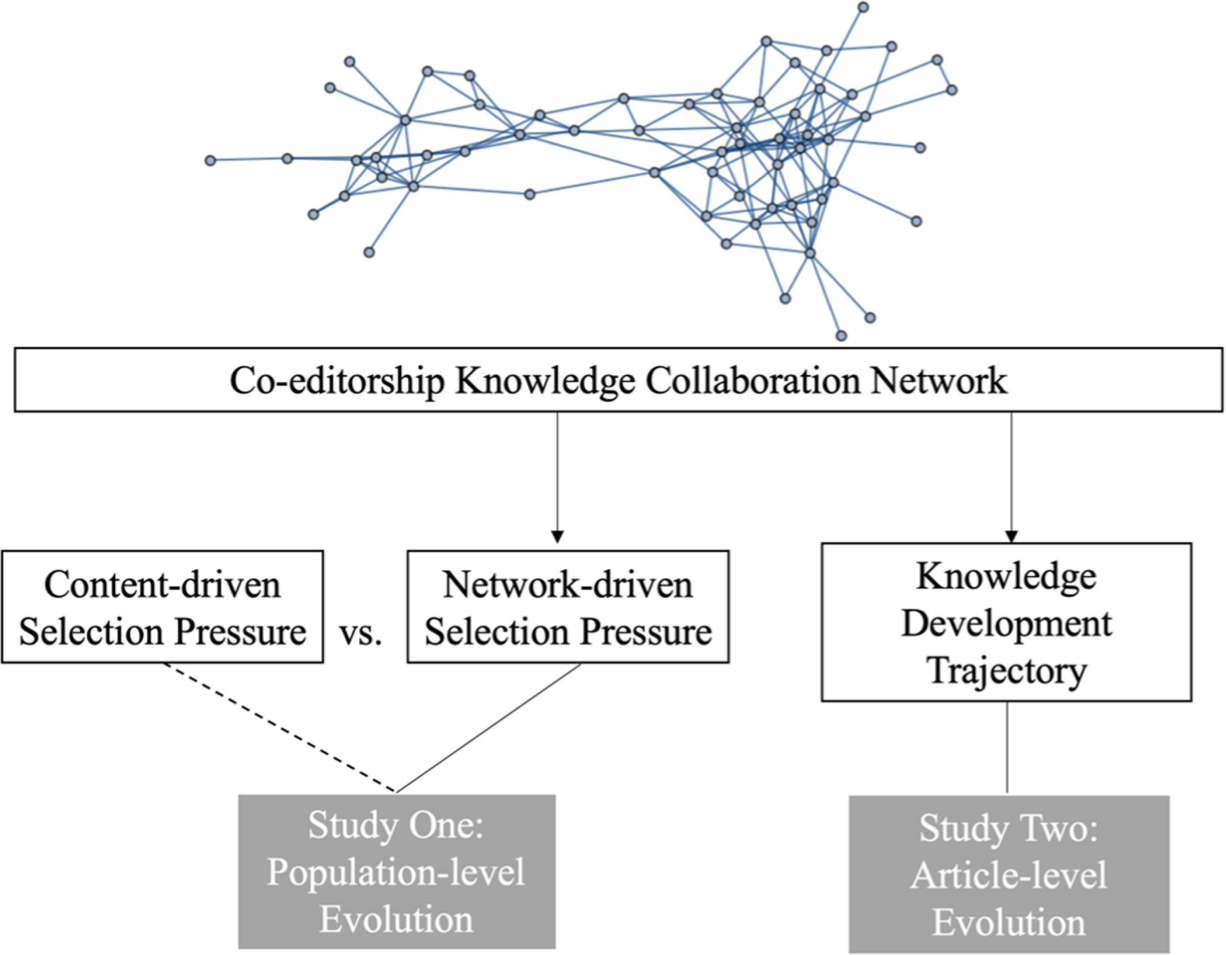
The conceptual model.

The current article includes two parts of investigation to systematically address the issue of whether and how network traits drive the evolutionary process of knowledge development, as shown in Fig 1. First, study one aims to pit network traits against content traits and observe which force is more potent in driving selection pressure. 21 different ways of partitioning the knowledge artefacts (11 content-based traits and 10 network-based traits) were used according to prior literature, and their relative importance in explaining knowledge adaptation outcomes were examined. This part would replicate and validate the hypotheses proposed in [7] about network traits’ significant role in driving cultural evolution. Results of study one confirmed a prior theoretical expectation that network traits play critical roles in identifying selection pressure, though not more so than content traits. In the second study, time series modeling was used to explain the findings of study one—*why* network traits matter in knowledge evolution. Drawing on the concept of artifact development trajectory [51], it found that editors’ collaboration networks may serve as information conduits and the network traits could consequently determine the extent of content exploration within an artifact’s feature space.

## 2. Study one: Network traits driving selection of knowledge artifacts

### 2.1 Content-driven or network-driven evolution?

“Neither genes, nor cells, nor organisms, nor ideas evolve. Only populations can evolve” [9]. This quote describes a classic idea in evolutionary theory that selection operates at the population level. Selection reflects the fact that some traits are more or less likely to be passed on from the ancestral population to the descendant population [10]. This view is helpful for explaining why social and cultural elements adapt over a trajectory that is non-random, without genetic evolution. In the context of knowledge production, certain traits of the knowledge content produced by a group of contributors at an earlier time point will be preferentially maintained (i.e., copied) to a later version, while the less desirable traits will be selected out (i.e., deleted or modified). Over time, people’s preferential selection of certain contents while discarding other contents result in the development of content in a knowledge collaboration system (Mesoudi, 2021).

A key issue in understanding the dynamics of knowledge evolution is to identify the cultural traits underlying cultural selection processes [6, 7]. In biological evolutionary processes, individual traits are often investigated as the defining indexes (*i*) that separate individuals into groups. Social science researchers face more ambiguities in defining traits but still provided several different ways to do so [11–13]. Cultural traits like norms and institutionalized habits [14], complexity of tools used in groups [15], food taboos adapting to local environments [16], and the opening moves used in Go matches [17] have been examined previously. A consensus is that the defining traits could be highly flexible, as long as they effectively distinguish certain cultural artefacts from others.

In the context of knowledge development, two major types of traits were thought to be critical—content-based traits and network-based traits, though their relative importance is unclear. Traditionally, information system scholars and computer scientists widely adopted the former perspective and distinguish knowledge artifacts based on the directly *observable content* of an informational product [5, 18–21]. They derived characteristics from textual contents and analyzed their differential impact on success in terms of viewership and popularity [5, 18–21]. This article uses the term “success” interchangeably with “fitness”, as the goal of knowledge development is often to have more people to read, remember, and use that information. For example, [5, 18] showed that length of the articles or edit length is positively associated with the quality of information. Kräenbring et al. [22] and Candelario et al. [19] both suggested that text accuracy and completeness are two important dimensions of medication-related content on Wikipedia. [21] suggested the verifiability of references across language versions to be a positive quality dimension of Wikipedia articles. [23] proposed eight dimensions when evaluating encyclopedia quality, including content scope, format, uniqueness, etc. [24] proposed content selection criteria such as credibility, importance, and plausibility. Beyond these lists, Wikipedia also provided its own list to guide users in evaluating article quality, which includes eight main criteria: well-written, comprehensive, well-researched, neutral, stable, correct and consistent style, media content with proper copyright status, appropriate length [25].

On the other hand, adopting the community ecology theory, Monge et al. [26] added a network-based interpretation to emphasize the role of network traits in driving the adaptation of cultural artefacts. They suggested that there are analytical gains if we move our focus from contents of a piece of information to also include the networks that this information is embedded in. They empirically validated the idea that network metrics can actually identify stronger selection forces, as measured by the Price equation [27, 28], than the traditional content-based approaches [7]. Specifically, they collected eight networked populations evolving over time, such as hyperlink networks of YouTube videos, organizational networks from the microcredit crowdsourcing platform Kiva, and the international trade network among 118 countries, and found that network-based characteristics can identify stronger natural selection than content-based characteristics in most of the empirical contexts. Importantly, this empirical study concluded that network traits identify stronger selection pressure, more so than the traditional content-based traits.

Other researchers, though not explicitly using evolution terms to guide their analyses, also emphasized the importance of analyzing the social graphs generated by social interactions to understand why a piece of content could achieve success. For example, [29] found that the relative position of an article within the collaboration network predicts Wikipedia content quality. La Robertie et al. [30] designed algorithms for calculating Wikipedia article quality score, which confirmed the idea that knowing the authors’ network positions can effectively predict article content quality.

However, open knowledge collaboration systems may challenge the general claim that sociocultural evolution is network driven. Though [7] suggested network traits drive the evolution, a close reading of their findings indicates their conclusion may not apply to knowledge collaboration. First, knowledge artifacts largely succeed based on content, suggesting a counterexample. Among eight datasets used in [7], only YouTube’s PBS video hyperlink network—an informational community—contradicted their conclusion. Knowledge artifacts may depend primarily on their content, not network characteristics, to spread and persist. As the sole exception, YouTube’s informational community shows content may better explain selection in such contexts. Second, their findings require further empirical validation. My analysis found that 76–77% of their pairwise comparisons (that is, combining all eight datasets as one) reported were statistically insignificant, indicating no difference between content- and network-based selection pressures for most comparisons. This lack of consistent evidence also necessitates further replication in the context of knowledge collaboration systems.

Thus, study one asks:

In knowledge collaboration systems, do network-based characteristics indicate stronger selection pressure than content-based ones?

### 2.2 Measuring selection pressure with the price equation approach

This section presents the Price equation approach which allows for comparison of the strength of selection forces for different population partitioning method. Price [31] introduced an “exact and complete” mathematical description of evolutionary change [32]. It describes how any measurable trait *z* (e.g., body length, finger number, to degree to altruism) changes from one generation to the next. Owing to its high generalizability, this equation has been equally employed in biological and cultural evolution [33] and remains a relevant explanation of the core principles in cultural evolutionary theory [34].

The Price approach argues that for the change from a previous generation to the next generation, there are two sources of evolution as described by two terms in the equation: the selection term (describing the connection between a trait change and fitness change), and an average transmission (describing an expectation value of the average trait change over generations). In most real-world examples, the knowledge development trajectory could be simultaneously determined by both actors. Biased selection or biased *reconstruction*, is a result of individuals modifying and transforming cultural traits in a non-random way based on their personal perceptions or cognition [35]. When an artifact was modified by several individuals (generations) in the same direction, the development trajectory will quickly converge on this individually favored path. The path is biased in that people do not make random decisions–decisions are always based on inductive reasoning, experience, trial and error learning with the goal of moving this reconstruction trajectory toward a more desirable direction, though the actual outcome remains uncertain. Transmission error was the unintentional change occurred when making the above move, such as the inevitable variation in oral communication of folklores. Prior studies often had to model the two simultaneously, or create a lab setting to closely focus on the unbiased reconstruction effect [15]. A big advantage of using knowledge collaboration systems as our research context is that its high fidelity in digital knowledge creation helped to isolate the effect of transmission error from selection pressure [36]. This context allows easy access to data of biased content reconstruction in a large-scale non-laboratory setting, an improvement compared to prior efforts [36]. It also means we could adopt the simplified version of the Price equation by only focusing on the selection term.

A simplified Price Equation (for more complex versions, see [28, 32]) proposed to measure selection pressure to be:

$$\Delta \bar{w}\;=\;\frac{var(w_{i})}{\bar{w}}.$$ (1)

Essentially, the right side of the equation refers to a mean-normalized variance of trait (*i*) fitness. Change in average population-level fitness $\left(\Delta \bar{w}\;\right)$ is thus a result of the variance of subgroup trait fitness. Certain traits are adopted or not adopted by group members, and as a result their relative distribution in the population changes over time [37]. When we can observe direct inheritance link between a cultural trait and a change in fitness, *w*_*i*_ can be interpreted as the payoff (number of offspring) of the cultural trait at a give time [38]. To take an example, the major of Architecture was at some point a popular choice among Chinese college students, and had then a high fitness (*high w*_*i*_), but it subsequently got out of fashion, and now has a lower fitness (*low w*_*i*_). And $\bar{w}$ is calculated as the population offspring at time *t* + 1 divided by the number of ancestors at time *t*.

Based on the above equation, selection effect arises from the fact that different subgroups within a population have differential reproductive rates, and there exists no mutation bias which otherwise may confound this equation. The “better” traits help the population to move toward a direction with higher fitness value. Overall, higher value of the equation indicates that a partitioning method (i.e., way of defining *i*) attracts more selection pressure and will more effectively differentiate advantageous members from the less advantageous ones [27]. When there are several feasible ways of partitioning the population, comparing the selection forces identified by each partitioning method will illuminate which trait is the most important features that identify the fitter individuals from the less fit ones. Next, two major ways of identifying the subgroups in a population of knowledge artefacts are introduced.

### 2.3 Network traits

Three important network structures–network embeddedness, network connectivity, and network redundancy—will be considered as potential network traits driving knowledge evolution. They are commonly used network structural constructs in prior research of online knowledge systems [5, 29].

First, in the context of knowledge creation networks, researchers have used embeddedness, the extent to which a particular piece of content is connected to other pieces of content through the network of content creator, as a proxy for the centrality in information exchange [39, 40]. Higher embeddedness means the artefact may be holding a key position because many other contents are related to this article, or because this artefact is often on the connection path among other contents [40, 41]. So, contents with higher embeddedness receive more attention and resources from the contributors and can potentially receive richer or better information resources as contributors bring their learning and experiences accumulated elsewhere to contribute to the focal content. Seven metrics from a family of centrality measures are used to describe embeddedness, including *degree centrality*, *closeness centrality*, *betweenness centrality*, *eigenvector centrality*, *PageRank*, and *hub*.

Connectivity of an individual artifact’s local environment is another important network trait. A highly connected or clustered local neighborhood means that the neighboring contents are themselves well-connected. It means that information can flow smoothly and efficiently in this neighborhood, without being dominated or controlled by a few elite nodes. This is often a structural signal of highly efficient information exchange pattern [42]. The focal piece will certainly derive advantages by being part of this highly connected neighborhood, and it can also be a well-connected information exchange hub serving the local network by closing triangle networks or by closing cliques. Having a highly connected local environment (local network level) or being in a position that facilitates connectivity (node level) can bring more resources to the focal artefact, and thus makes it more likely that the article will be useful for the readers. *Clustering coefficient* and *density* were two metrics used to describe the connectivity of a local environment—defined as a focal article’s two-step network neighborhood. The step of two is chosen because this will lead to a network that is large enough to have a meaningful number of possible connections, and small enough to remain relevant for the focal node.

Redundancy of an article’s local environment can be another important factor that impacts how much new and unique information that article has access to. If embeddedness is describing how much information resources are available to a focal article, then redundancy of an article’s local environment determines how much of those resources are truly unique and can contribute meaningful new content to the article. The value of encyclopedia entries partly hinges on the extent to which they can provide unique information to readers. Imagine an article that occupies a highly embedded position in a highly redundant local neighborhood, the amount of total information available to the article may be high, but the amount of new and unique information would still be low. In this sense, it matters that an article has access to diverse and rich information resources in a local neighborhood so that the content presented is truly unique and useful for the readers. This means that a non-redundant local neighborhood could help an article to become more fit. Redundancy can be measured in two different ways, *constraint* [43] and *effective network siz*e [44, 45]. Note that higher values of effective network size indicate less redundancy while higher values of constraint indicate more redundancy.

### 2.4 Content traits

The current study will also consider some previously identified content traits as defining characteristics of subpopulations, to explore whether and how they are driving evolutionary dynamics of knowledge development. These characteristics were mainly based off a list suggested by [20] with some modifications catering to the Wikipedia site. This proposed list has several advantages, including (a) it reaches a balance between number of characteristics and prediction accuracy, (b) the characteristics are highly interpretable and meaningful, and (c) this list has been validated multiple times by both industry practitioners [46] and other researchers [47]. These 11 content-based characteristics are categorized into five groups based on their theoretical relatedness, as explicated in Table 1.

**Table 1:** List of content-derived traits.

Type of trait	Measure	Description	References with Empirical Evidence
Scope of content	content length	Article length in bytes	[8, 18, 20, 40, 48, 49]
	image by length	Number of images / length of article in bytes	[20, 48]
External references	number of references	Number of references	[18, 20, 40, 48, 49]
Indexing	number of page links	Number of links to other Wikipedia pages	[18, 20, 40, 48, 49]
	number of categories	Number of categories tagged in the text	[18, 20, 40, 48, 49]
Formatting	number of cite temp	Number of citation templates	[18, 20]
	has infobox	A binary indicator of whether it has infobox or not	[18, 20]
	number of lv2 heading	Number of level 2 headings	[18, 20]
Readability and clarity	flesch reading score	= 206.835-(1.015 * avg_sentence_len)–(84.6 * avg_syllables_per_word)	[20, 48]
	coleman liau index	= 5.88 * avg_word_len -29.6*avg_sentence_len– 15.8	[18, 20, 40, 49]
	difficult words	Number of words that do not appear in a list of 3000 common English words that fourth-grade American students can reliably understand	[20, 49]

### 2.5 Wikipedia as a research site for knowledge evolution

As an example of online knowledge collaboration communities [4], the free encyclopedia Wikipedia probably constitutes the most well-known collaborative system where any user can create and edit knowledge content [30]. In this peer-to-peer content creation paradigm, users are able to contribute and nurture collective intelligence by adding, revising, and deleting small chunks of information, ‘wiki’, which eventually becomes a part of collective intelligence in the knowledge ecosystem shared by all internet users [3, 50]. Collective knowledge creation occurs when the cumulative efforts of users are integrated together through internet-based technologies. Prominent examples of this type of organization include Wikipedia, StackOverflow, GitHub and Kaggle. Many scholars have discussed these knowledge collaboration systems, from the aspects of information system management [8, 51, 52], but the evolutionary nature of knowledge development exhibited on these platforms was seldom discussed [53].

This article considers online collaboration systems to be an interesting venue to observe knowledge evolution manifested in forms of collective writing and editing. For example, the Wikipedia platform takes information generated elsewhere as input, and it delivers encyclopedia content as output to users. Imagine one piece of scientific finding that has been listed as a Wikipedia entry. The specific way of presenting this information could still go through many rounds of writing and editing before it achieves a stable and mature status. Sometimes the process never ends, because the explicit verbal representation of meanings is in itself a contingent and fluid process, and only an approximate resemblance of the actual information [54]. Editors could always add new content to enrich this information and they could always modify the content to improve its clarity. Thus, different ways of language representation will lead to different outcomes for these encyclopedia items. This article focuses on the evolution of knowledge observable on such a collaborative knowledge system, where millions of editors help with the content development by searching for most appropriate knowledge representations.

### 2.6 Data collection and network construction

This study analyzes the WikiProject *Aquarium Fishes* as the population of interest. A WikiProject is comprised of a group of volunteer contributors who commit to develop and organize articles related to a focal topic, such as medicine, fashion, history, etc. [38]. Overall, the English Wikipedia currently has over 2,000 WikiProjects. Choosing a WikiProject, instead of randomly choosing articles from Wikipedia, made sure that the collaboration network can be meaningfully constructed, as editors are expected to have engaged in knowledge exchange in the same topic area. Otherwise, the network would be too sparse with little tie connections. For each article in this sample, the following information were collected: (a) complete editing history; (b) editor information; (c) fitness measures.

Specifically, choosing the WikiProject *Aquarium Fishes* has two advantages. First, its initial size of 910 articles is both large enough to provide rich information while small enough to be handled by network analysis techniques. Second, it is also an area that is not overly active or overly popular, compared to socially sensitive topics. It does not receive fluctuating attention by the “fluid” or one-time editors who are drawn by sensational news and leave when the entries are no longer popular [55]. The internal dynamics of its editors are relatively stable, which is ideal for our research focus on the internal dynamics generated by editors’ network behaviors rather than the momentum brought by external environment shocks. (S1 File provides detailed description of the page view dynamics to confirm this point).

**Fig 2:**
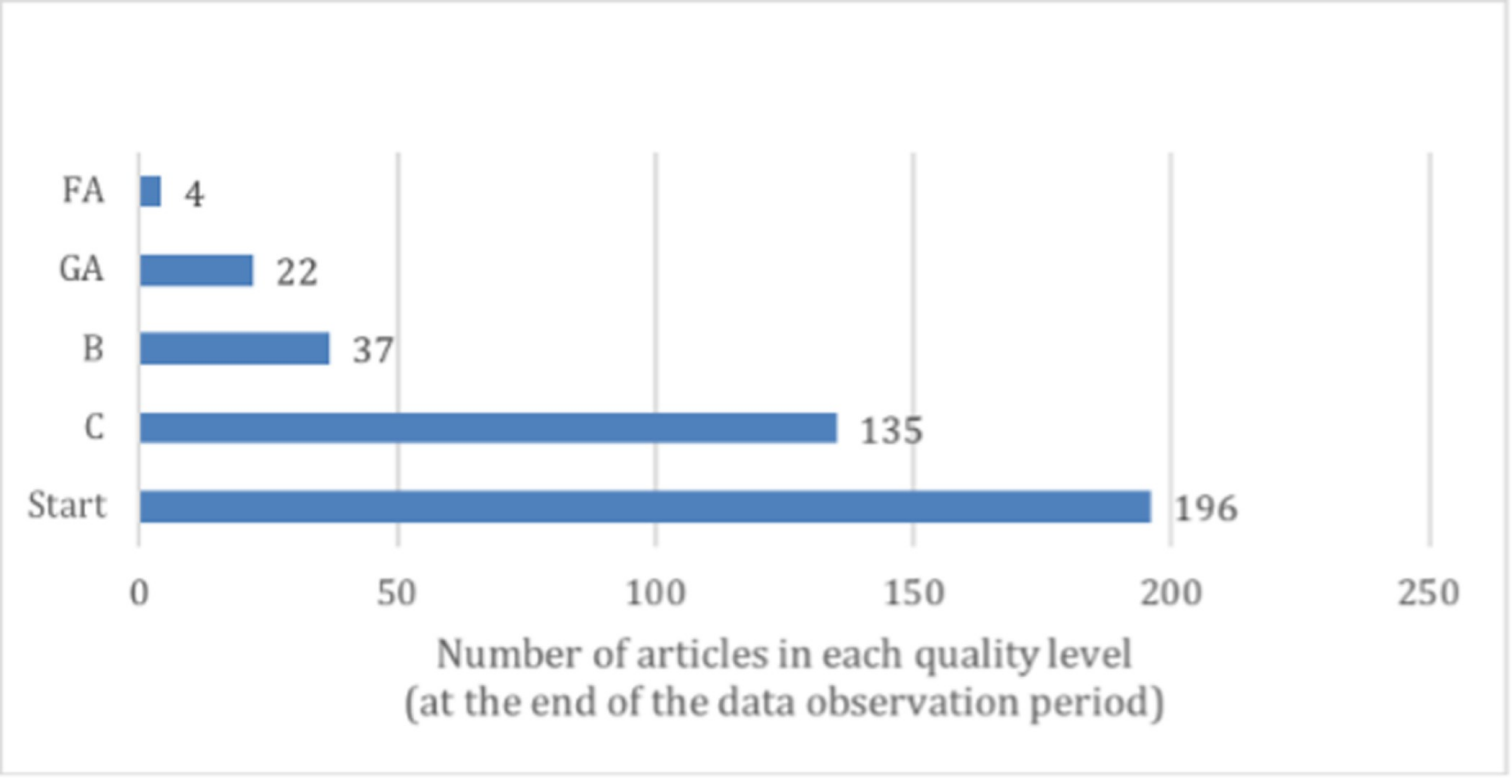
Number of articles in each quality level. The X-axis represents number of articles and Y-axis represents the quality levels from low to high, where Start is the lowest level and FA the highest quality in the dataset.

Among the initial set of 910 articles in the WikiProject of *Aquarium Fishes*, the following exclusion rules were applied: (1) articles that were not evaluated, or for some reason do not receive a valid evaluation score; (2) articles that were created after January 1, 2017, the beginning of the data observation period. A critical variable “page views” was made available only after this date. These two exclusion steps led to a final dataset of 394 articles. Fig 2 shows the distribution of the articles in each quality level (*Start* being the lowest and *FA* being the highest quality). 196 out of the 394 articles (49.7%) belong to the Start level, which is the minimal quality level collected in this dataset. Data collection was completed in February 2020.

The current project considers the co-editorship network among knowledge contents. This co-editor network is converted from a bipartite article-editor network that captures how human actors (editors) can potentially exchange information, knowledge and resources among different articles for the purpose of knowledge creation. In the co-editor network, nodes are the individual articles, and each tie represents the co-editing behavior of an editor who worked on both articles. This network is an undirected (sharing an editor is not a directed link) and unweighted (not considering for multiple co-editors). The decision to disregard edge weights was driven by the fact that edge weights may not always be applicable to the extensive range of network metrics we are considering. By doing so, we can quickly explore the fundamental structure of the network. The structure of the co-editor network reflects the how articles are embedded within a population of articles while the editors exchange information, knowledge, and experiences among these articles. The co-editor network is also a commonly studied network type in prior literature about knowledge creation systems (Qin et al., 2015; Kane and Ransbotham, 2016).

Additionally, leveraging the availability of Wikipedia edit history with timestamps, the longitudinal dataset was divided into weekly observation windows. The dynamic dataset includes 163 weekly observations in total. The choice of a ’weekly’ interval strikes a balance between daily and monthly observations—it is long enough to yield discernible selection outcomes and, at the same time, short enough to capture the rich temporal changes in the values of these traits. For each week, the network was constructed by collapsing (cumulating) all linking activities within that week into a single slice. This means that co-editorship ties established on different days within the same week are considered valid links in the weekly network. By opting for a cumulative weekly network instead of an instantaneous snapshot at the end of each week, we effectively captured and fully utilized the linking activities occurring over time. (The above description about the nature of the temporal collaboration networks apply to both studies. For brevity, method section in study two does not repeat this description).

### 2.7 Fitness

Though there are rich possibilities of interpreting what “success” or “fitness” in Wikipedia means, this project focuses on viewership of the article. This measure is close to the idea that evolutionary fitness is generally represented by replication, or the process of making more replicates (i.e., copies, offspring) of the piece of information. To say that a Wikipedia article acquired “numerous” copies in readers’ consumption processes would indicate that the article content has achieved success in spreading to a wider audience. Since Wikipedia is generally transmitted online, the spread of an article could be roughly measured by the change in page views of articles. Higher page views indicate fitter content. This measure has been used by previous research as a measure of Wikipedia articles’ market value [40].

### 2.8 Findings

**Fig 3:**
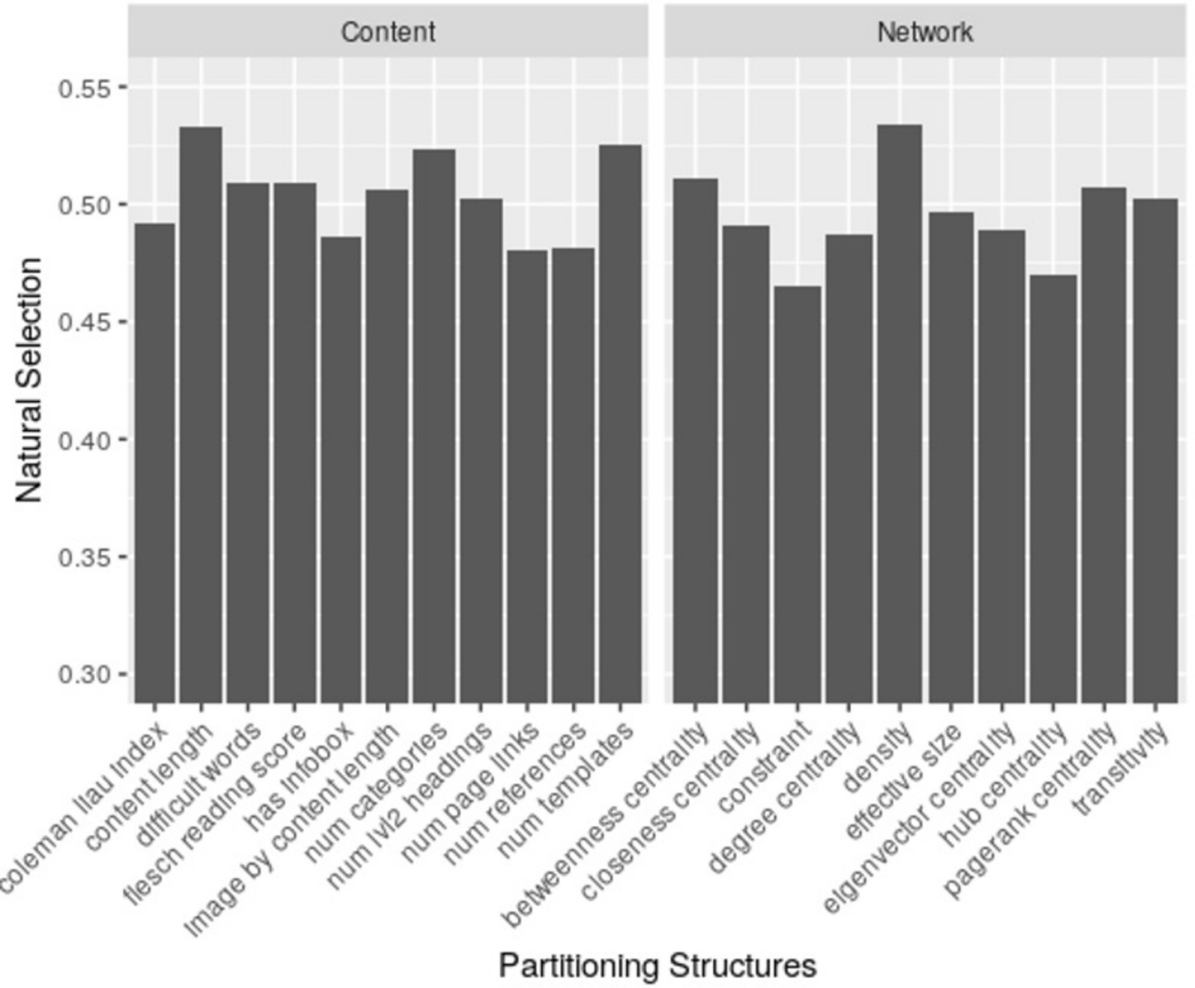
Average percentile rankings of selection forces based on each different partitioning structure. X-axis represents each different population partitioning structure, Y-axis shows the average percentile ranking of selection value. The “Content” panel on the left side includes the 11 content-based characteristics, and the “Network” panel on the right side includes the 10 network-based characteristics.

The dynamic dataset includes 163 weekly observations, since selection effect is based off changes between two generations, this dataset generated 162 valid selection pressure values. The raw values were then ranked by percentiles from the lowest to the highest, with lower percentiles representing smaller selection effects. The ranking treatment follows [7] and it is useful in removing the absolute value variances of selection effects while keeping the rank order. This also makes the results reported here consistent and directly comparable with prior studies. Together, the data set includes 21 partitioning traits (11 content-derived and 10 network-based ones) thus lead to 21 vectors of selection pressure values (vector length = 162) that reflect the evolutionary forces imposed by changes in each of these characteristics. Given that most of these trait values were originally continuous, proper adaptation for use in the Price equation involved binarizing them into high versus low value groups using a mean-based split. This data conversion step aligns with the methodology established in [7], ensuring consistency with previous practices. Fig 3 presents the average percentile rankings from two groups of traits.

The average content-based selection pressure is 0.504 and the average network-based selection is 0.495. The difference seems to be not large. Beyond eyeballing the differences in two groups, a Fisher-Pitman permutation test was used by building randomly sampled distributions of the selection effects, controlling for weekly observation periods.

To obtain the statistical significance of the comparison between these two vectors of selection pressure, an empirically generated sampling distribution will be created [56, 57]. To construct the sampling distribution, at a given time t, one value is randomly drawn from the (11 possible) content-based values and another value is randomly drawn from the (10 possible) network-based values. As such, each pair of data constitutes a randomly selected content-based trait and a randomly selected network-based trait at the same time. This procedure was repeated 10,000 times to generate 10,000 paired vectors for comparison. The Fisher-Pitman test, a nonparametric counterpart of the F test in one-way ANOVA, was used to analyze whether content-based or network-based characteristics are generally more powerful in driving the evolutionary change. The procedure will be implemented using the R package coin [58].

The result showed that the observed value is likely to happen in 23.83% of all random simulations (2383 cases out of 10,000 simulations, two-sided test). In other words, 23.83% of the simulations might generate more extreme values (including both greater or lesser) than the observed one, and 76.2% of the simulations generated less extreme values than the observed one. With *p* = .24, the content-based versus network-based traits did not drive significantly different evolution outcome.

Furthermore, the differences among the three types of network traits can be investigated, including network embeddedness, network connectivity, and network redundancy, using a similar comparison procedure. A Fisher-Pitman permutation test was conducted to examine if there is at least one group of values is significantly different from the others, controlling for the weekly observation period. Fisher-Pitman permutation test can handle more than two levels for an explanatory variable [59].

The result showed that the observed value is likely to happen in 1.21% of all random simulations (121 cases out of 10,000 simulations, two-sided test). That means, only 1.21% of the simulations might generate more extreme values (including both greater or lesser) than the currently observed one, and in over 98% of the random simulations we might observe less extreme values than the observed one. Specifically, the mean of connectivity type is 0.52, the mean of embeddedness type is 0.49 and the mean of redundancy type is 0.48. With *p* = 0.012, the omnibus null hypothesis was rejected. At least one type of network trait was significantly different from others in driving knowledge evolution.

The pairwise comparison between embeddedness and connectivity (*p* = 0.034), the pairwise comparison between connectivity and redundancy (*p* = 0.015), and the pairwise comparison between embeddedness and redundancy (*p* = 0.20) helped to determine which set is most significantly associated with selection pressure. The Bonferroni-Holm procedure [60] was adopted to evaluate the significance of *p*-values generated from each comparison. The findings show that connectivity-driven selection is significantly different from the other groups, while the difference between redundancy and embeddedness group was not significant. Taken the results together, the three groups of network traits can be ranked in the order of connectivity > embeddedness = redundancy.

Overall, network traits did not identify stronger selection pressure compared to the content-based traits. Both types of traits seem to be equally useful in driving knowledge evolution. Among the three types of network traits examined, those describing network connectivity was the most influential ones.

## 3. Study two: Networks traits driving knowledge artifacts’ development trajectory

### 3.1 Theoretical background

Study one found network characteristics can be important for knowledge selection outcome, assuming that collaboration networks reflect patterns of information exchange and thus affect knowledge growth. Study two then explores this implicit link between collaboration network structure and the growth trajectory of informational artifacts created.

An artifact’s *knowledge growth trajectory* [51] reflects the path that many contributors collectively develop a knowledge artifact by exploring different possibilities in a *feature space* (also called *solution space*). In various fields like design, engineering, and computation, the design of an artifact is represented by positioning it in a feature space, where the dimensions of the space define the various features of the artifact. Knowledge growth thus involves a collective and exploratory process of searching a wide range of possibilities in the abstract feature space and converging on a final solution, which is often conceptualized as collective problem-solving [42, 61–63].

**Fig 4:**
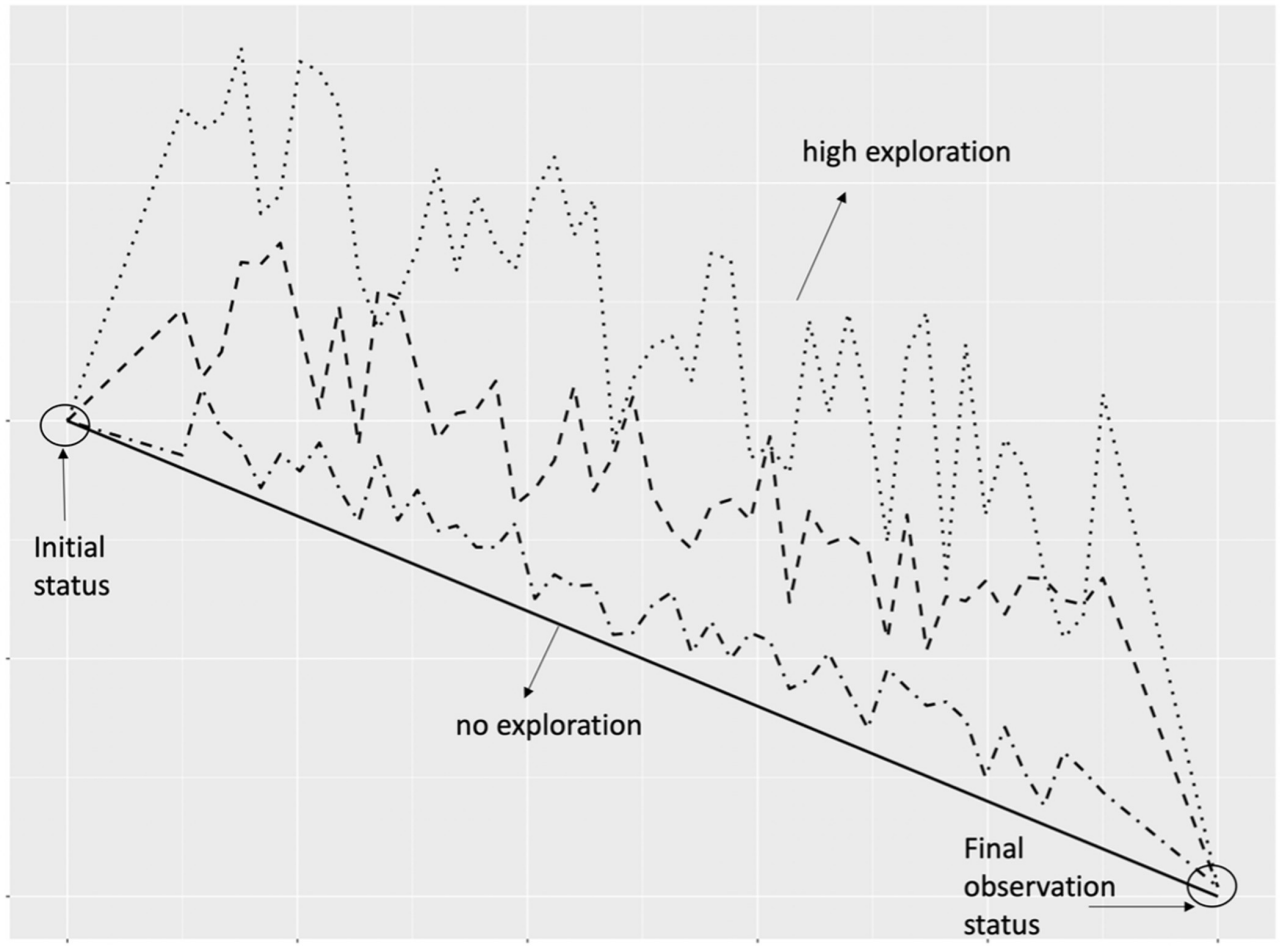
Conceptual illustration of artifact development trajectory and content exploration. The straight diagonal line shows a benchmark of no exploration. The artifact’s development path stays close to the desired direction with any deviation. Other three lines show increasingly explorative trajectories as the paths deviate from the benchmark line (no exploration) further. All lines have the same initial status and final observation status, which means that after different search processes, all artifacts arrived at the same final status.

The level of exploration of an artifact’s trajectory can be formally measured as the extent to which it deviates from a straight line connecting the initial and final version of the artifact [51]. High level of exploration occurs when the trajectory wanders through various design configurations that are later abandoned. Low level of exploration refers to when an artifact’s trajectory evolves in a relatively straight line. In the extreme case, no exploration occurs, and the artifact evolves in a directed manner without any trial-and-error. However, this scenario often fails to uncover new opportunities and innovative directions. Fig 4 illustrates the concept of exploration in development trajectory. Note that although the data collection occurred within the same "period of time" on the calendar, the articles were not at similar statuses at either the starting or ending points. Wikipedia articles are created and developed by volunteer contributors based on their own interests and availability, without a central organizational structure. Each article follows its unique development track, experiencing different stages of progress. The dataset’s composition highlights the varying development stages of cultural artifacts.

In online information systems, the artifact development trajectory emerges organically from contributors’ negotiation of different perspectives and the clashes of divergent opinions [51]. Thus, the trajectory is typically influenced by the extent of information exchange among contributors, which can be observed in the structure of their collaboration networks. In most feature space search processes, resource constraints (labor and time) limit the level of exploration that can be realistically achieved, so collective problem-solving typically invites users to adopt a heuristic approach to finding an acceptable solution. On Wikipedia, this search process unfolds as individual contributors pulling an article’s trajectory towards their individual habitual contributions. These pulls may stem from unintentional manifestations of the person’s preferences, skills, and expertise or deliberate attempts to shape the artifact according to their personal vision. When multiple contributors are entrenched in their preferred paths, they tend to pull the artifact in different directions, resulting in extensive negotiations and high exploration. When contributors can reach consensus regarding the desired solution, they reduce the level of exploration and converge on a collectively agreed-upon path quickly. Prior research has empirically shown that the working group characteristics (such as whether members remain working for the same article and whether members are registered community members) impact the level of exploration in Wikipedia development trajectory [51].

The notion of development trajectory from information system scholarship fits with several related concepts used in cultural evolution literature. First, the guided variation model of knowledge development [35] proposes that knowledge develops along a non-random path. This means that variants of knowledge seem to develop in a "linear" direction not because people have pre-planned the path, but rather through trial-and-error learning. A previous generation of an artifact passes on desirable traits to the next modified version, pushing forward the evolutionary process. If every contributor develops traits in the same direction, then the population quickly converges on this individually-favored trait. Otherwise, different individual preferences lead to a more rugged and inconsistent knowledge trajectory. Second, the theory of cumulative cultural evolution (CCE) describes a problem-solving phenomenon at the population level over time [64]. It involves the selective learning of adaptive information that results in the gradual accumulation and the development of cultural traits that are beyond individuals’ inventive capacities. This process leads to the repeated modification and social learning of behavioral traits over successive generations, resulting in cultural traits that improve in efficiency, such as fitness. With CCE capacity, human beings are able to solve complex challenges by developing sophisticated knowledge. Generally, evolution theorists believe that the cause of knowledge change lies within the individual’s cognition and perception of the traits they modify. Considerable scholarly attention has been given to laboratory experiments exploring individual cognitive tendencies towards adopting, choosing, modifying, and disseminating knowledge [65]. However, these experiments do not fully capture the complex social dynamics that shape knowledge development in the real world. Knowledge development occurs within a social context, where information interactions are critical to shaping the trajectory of knowledge development. Laboratory experiments have difficulty modeling these collective-level dynamics.

To address this limitation, the current study drew on both information systems research and evolutionary theory to examine the dynamic relationship between contributors’ informational interactions (using a social network lens) and the knowledge development trajectory [42, 51, 66, 67].

### 3.2 Hypotheses development

There is ample evidence in literature showing correlational connections between network configurations and system-level knowledge outcomes [8, 42, 67, 68], but there are few that use longitudinal design to observe the dynamic impact of network configurations on knowledge evolution [69]. Adopting a new approach for quantifying text-based content exploration, this study suggests that network configurations are significant predictors of knowledge artifacts’ subsequent development trajectory [51].

Explorative search is essentially a recombination process. Knowledge development arises as the result of recombining and transforming existing and novel elements of knowledge into something new [70–72]. Thus, the social structure in which individual actors are connected and embedded heavily impacts the exploration outcome. The three types of network structure of interest in this article, including network embeddedness, network connectivity, and network redundancy, have been shown to be associated with key knowledge outcomes, as each one facilitates or prohibits information exchange in different ways.

#### 3.2.1 Network embeddedness and exploration

Embeddedness refers to the structural characteristics of having a highly accessible and central position in a network. There has been a historical link between network embeddedness and desirable social and economic outcomes [41, 73] because actors with high embeddedness are in advantageous positions for knowledge acquisition, transfer, and exchange, thus facilitating effective knowledge development and innovation. Ample evidence in the setting of small-and medium sized enterprises [74, 75], R&D project network [76], service industry [77], bio-pharmaceutical industry [78], technology alliance network [71] consistently showed that high network embeddedness is associated with better knowledge development outcomes such as new product development or innovation performance. Beyond enterprise-level research, individual actors are also found to greatly benefit from positions of high network embeddedness. A large-scale web experiment found that Facebook users who occupy central positions can insert higher influence on their peers as the information they disseminate are more likely to be adopted by their social contacts [79]. Scientists’ network embeddedness also positively predict their publication output number [80]. Thus, consistent evidence supports the idea that position in the network affects the knowledge exchange and development opportunities of an actor. Having high levels of interaction with network members increases information sharing, a key condition for effective knowledge exchange and search process [78, 81]. Rich possibilities of leveraging network partners as information sources allow an actor to more thoroughly explore the solution space in the process of knowledge search and development.

Thus, it is hypothesized that:

H1: Over time, *higher* levels of network embeddedness in a knowledge creation system will lead to subsequently higher levels of content exploration in the evolution trajectory.

#### 3.2.2 Network connectivity and exploration

Network connectivity, the degree to which network actors are connected to each other, greatly affects the speed and efficiency of information flow within a network [42], which in turn, can impact knowledge development and problem-solving. The positive and negative effects of network connectivity on information exploration have been investigated by different scholars using various network configurations and search outcomes.

A general conclusion is that network connectivity plays a key role in knowledge outcome, but its impact varies. [67] found that a high clustering coefficient in a network promotes exploration in the information space, as it helps the spread of novel information. However, it inhibits exploration through the solution space, reducing the diversity of knowledge created. [42] used computer simulations to show that a highly connected network positively affects information diffusion, which may facilitate the spread of effective strategies. However, an inefficient (poorly connected) network maintains diversity of information in the system and is thus better for exploration than an efficient (well-connected) network in the long term. For intermediate time frames, there is an inverted-U relationship between connectedness and performance, in which both poorly and well-connected systems perform badly, and moderately connected systems perform best. Similarly, Gilsing et al. [71] found that a medium level of density was optimal for technology alliance networks. High density inhibits the existence and utilization of diversity and novelty value, while low levels do not support knowledge absorption. Mason and Watts [66] conducted web experiments by manipulating different team structures and showed that those networks with better connectivity (i.e. shorter path lengths) performed significantly better than those poorly connected. Efficient networks outperformed inefficient networks for two reasons: first, because information about good solutions spread faster in efficient networks and, second, because, contrary to theoretical expectations, searchers in efficient networks explored more, not less, than those in inefficient networks.

Overall, the impact of network connectivity depends on the type of search task (i.e., information space or solution space) and time frame. High connectivity can facilitate information diffusion, leading to the rapid convergence of ideas and reducing the level of exploration. On the other hand, high connectivity allows for fast spread of information and creating more opportunities for different solutions to be explored and learned. A review of literature thus leads to two alternative hypotheses:

H2(a): Over time, *higher* levels of network connectivity in a knowledge creation system will lead to subsequently *higher* levels of content exploration in the evolution trajectory.

H2(b): Over time, *higher* levels of network connectivity in a knowledge creation system will lead to subsequently *lower* levels of content exploration in the evolution trajectory.

#### 3.2.3 Network redundancy and exploration

Network redundancy is the extent to which multiple connections exist between actors within the network. High redundancy means that there are many redundant ties or connections within a network, thus the same information or knowledge can be accessed through multiple paths, and that there is a high degree of overlap in the information available to different individuals or groups within the network. This redundancy can help to facilitate communication and coordination but may also limit the diversity of information available to individuals. Research shows that access to non-redundant information and diverse perspectives is critical for both individuals and firms to explore more information and perform well.

At the individual level, people with contacts (ego-network) who are not themselves connected can access multiple sources of non-redundant information, and this can be useful in encouraging their colleagues to become more innovative [82]. Thus, low network redundancy is beneficial for the likelihood of being a catalyst of innovation. Similarly, [80] examined how redundancy affects scientists’ research quality and quantity. Non-redundant information helps with better research citation outcomes, suggesting that network redundancy may hinder content exploration in scientific research.

At the firm level, businesses also benefit from having access to non-redundant information to fully explore innovative solutions. [83]‘s research on European agri-food firms found that bridge ties, or the number of diverse network memberships, facilitate innovation, and the diversity of partners increases the innovation output of firms. [84] studied venture capital firms network and revealed that network redundancy is overall a negative predictor of firm performance, further supporting the argument that lower levels of network redundancy may lead to higher levels of information exploration. Finally, [85] identified that the main effect of tie weakness for problem solving is significant and positive. Teams with more weak ties shorten task completion time when the knowledge to be transferred is codifiable.

The above discussion thus leads to the third hypothesis:

H3: Over time, *lower* levels of network redundancy in a knowledge creation system will lead to subsequently higher levels of content exploration in the evolution trajectory.

**Fig 5:**
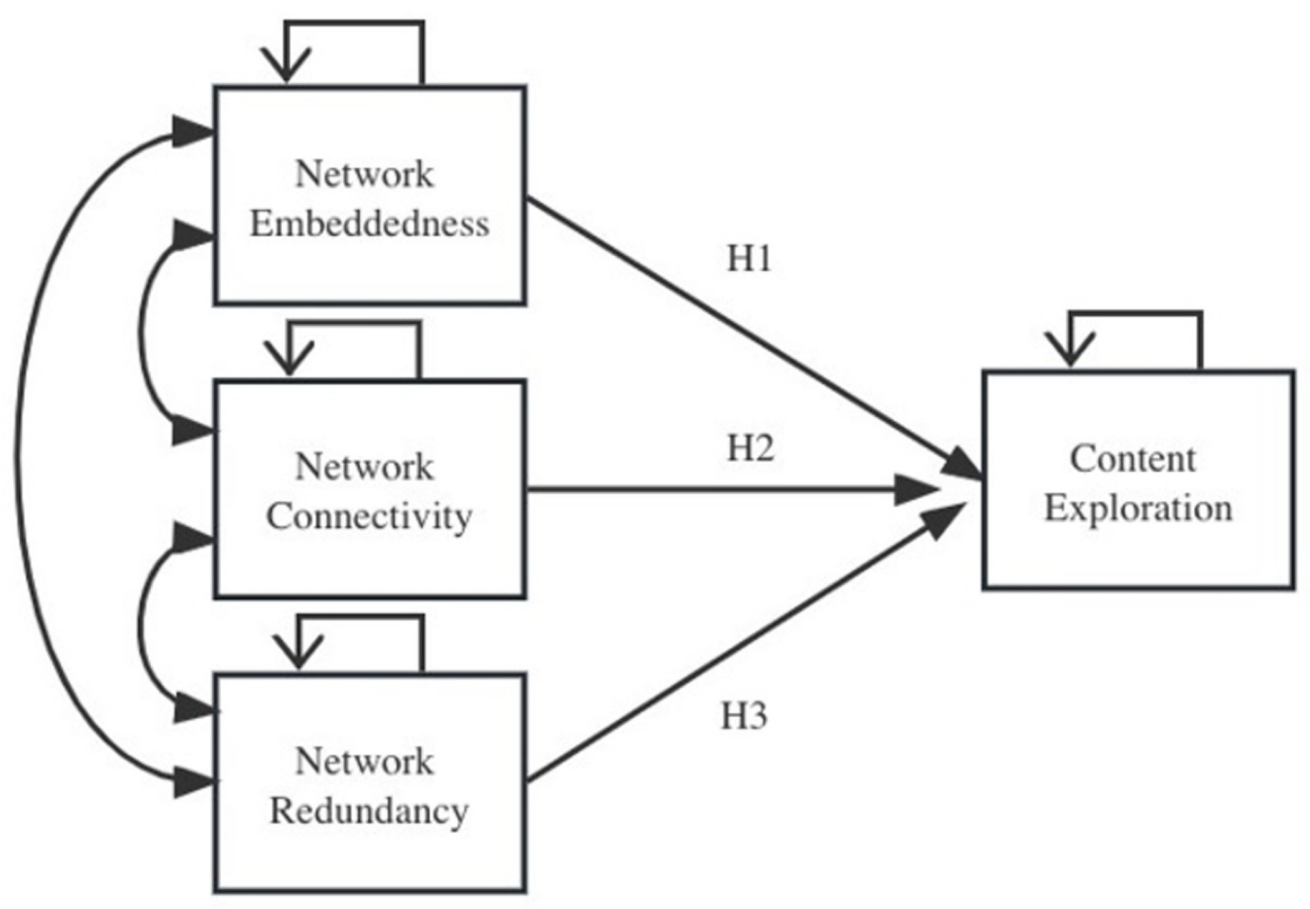
Theoretical model of network configurations that predict content exploration. The self-loops (on the top of box) represent a control of the influence of the variable’s history on itself. The curved arrows (on the left side of the boxes) connecting three explanatory variables represent the fact that the model will control for the influence of the other two explanatory variables, when regressing response variable on each explanatory variable.

A graphical representation of the hypotheses is shown in Fig 5.

### 3.3 Data and method

#### 3.3.1 Network-based characteristics

The three sets of network metrics used were the same as discussed in study one.

#### 3.3.2 Content exploration

The operationalization of content exploration was adopted from [51], inspired by the idea of solution searching in the areas of design and engineering. (A full explanation of the measurement of this construct can be found in S2 File). The space made up of all possible solutions is considered a feature space, and the process of surveying the space of possibilities is referred to as search [66, 86]. The end goal is for creators to identify a position in the space that leads to the optimal outcome, such as highest quality, lowest cost, etc. The decision to arrive at such a position is thus a search problem.

Content exploration, according to [51], was defined as the degree of which an article explores a two-dimensional feature space constructed by an article’s starting and ending position. At the beginning stage of content creation, there are many possibilities and many possible directions for the content to explore. The process of generating and evaluating a wide range of artifacts (positions in the feature space) is referred to as exploration [86, 87]. The realized positions of that artifact constitute a line or a development trajectory in this space, which is analogous to a regression line in a two-dimensional regression graph. An illustration of high versus low exploration in the evolution trajectory is given in Fig 4.

In the context of Wikipedia article creation, [51] suggested to model content exploration as a dynamic process that moves from a starting point to an end point in a two-dimensional space. The starting and the ending point connects a straight line–the benchmark trajectory. As an article gets created and developed, each version of the artifact traverses from one position to another. Positions that fall onto the baseline are considered to have zero exploration, because they move in a direction that they “should” go by not deviating from the baseline trajectory at all. Positions that are closer to the baseline trajectory are considered to have low exploration. Positions that are farther away from the baseline trajectory are considered to have higher exploration.

This measure of content exploration is naturally a longitudinal observation throughout a period of time. [51] used fixed effects models to control for the temporal factor by including two proxies of “time”, one is the edit-session number and the other is article age. It was a limitation of their work that their analysis did not establish a causal relationship between predictive factors and artifact exploration. The current study treats time-varying effects of knowledge evolution with more sophisticated time series analysis in modeling content exploration.

#### 3.3.3 Time series analysis

Transfer function (TF) models, a technique of multivariate time series modeling [88, 89], will be used to model the dynamic process of how network configurations cause changes content exploration [88, 90].

This is a dynamic regression technique that allows the explanatory variable(s), a dynamic process, to influence the response variable, also a dynamic process. This technique will describe how the changes in the explanatory variable get transferred to the response variable, by identifying a transfer function that is conceptually similar to the regression coefficient in classic regressions.

Formally, transfer function models can be written as

$$y_{t}=c+\beta_{1}y_{t-1\;}+\;\cdots \;+\beta_{p}y_{t-p}\;+\;\theta_{0}x_{t}\;+\;\cdots \;\theta_{q}x_{t-q}$$

where *β* are coefficients that reflects the lagged effects of Y series’ history on its current value, and *θ* coefficients reflect the lagged effects of X series’ history on Y. *p* represents the number of lags of the dependent variable to be modelled, and *q* denotes the number of lags of the exogenous variable to be modeled. In transfer function models, the “coefficient” is, instead of a single value, actually a polynomial that accounts for several past values of X. Each of these past values of X has different and time-varying effects on Y. If the parameter estimation shows that certain network configurations have significant lagged effects on content exploration, it can be concluded that network configurations indeed cause changes in content exploration levels over time.

#### 3.3.4 Benchmark ARIMA model

Before fitting an ARIMA model for the dependent series, a stationarity check is necessary [88, 89]. The raw series of content exploration was found to be a non-stationary series, judging by the Augmented Dickey-Fuller Test (*ADF* test statistic = 0.36, *p* = 0.29). A first order difference is enough to make the raw series stationary (*ADF* test statistic = -7.8, *p* = 0.01). ARMA (p,q) models for the differenced series can be constructed. As present in Table 2, ARMA model (1,1) is the best model among the alternatives judging by AIC and BIC values, and it generated an adjusted *R*^*2*^ value of 0.37.

**Table 2:** ARMA model comparisons.

p	q	Adjusted *R*^*2*^	*AIC*	*BIC*	Q-test *p* value
0	1	0.18	1.13	1.19	0.29
0	2	0.22	1.07	1.15	0.97
1	0	0.26	1.01	1.07	0.04
1	**1**	**0.37**	**0.88**	**0.93**	**0.67**
1	2	0.36	0.88	0.98	0.58
2	0	0.29	0.98	1.06	0.24
2	1	0.36	0.89	0.98	0.69
2	2	0.37	0.89	1.01	0.59

The bolded row shows the best model selected.

The input series left were then entered into the transfer function models to evaluate their specific effects on the dependent series [88–90]. The estimation used statistical software EViews 10.

#### 3.3.5 Lag selection

To determine the lag length (a lag refers to the difference between two weekly observations), for the dependent series, it can be reasonably assumed that most recent one or two lags of the dependent variable should have some impact on the current value. Thus, the maximum lag length of the dependent series was set to three to make sure the procedure evaluates all possible candidate lags.

For the input series, the maximum lag length was set to ten. This is a choice guided by previous literature. Ivanov and Kilian [91]‘s review paper suggested that lag length needs to correspond to realistic time windows in terms of data collection. For example, quarterly observations could select lag length of four or eight; monthly observation data could select lag of six or 12, corresponding to half a year or a year of lasting impact. Also, Johnston and Dinardo [92] suggested that it is better to start with a more complicated model and longer lag structure, and then move backwards. Thus, this study uses a maximum lag length of 10 to evaluate input series’ impact for the following two and a half months.

Next, stationarity was checked for each series and *ADF* tests for all the raw series could not reject non-stationarity at 5% critical value (see Table 3). Meanwhile, all first-differenced series are stationary at 1% level. These differenced series can be interpreted as “weekly change” of that variable from the previous week. First-order differenced data instead of the raw data are used to reduce correlations among lag terms of the input series.

**Table 3:** Augmented Dickey-Fuller test results.

Variable	Augmented Dickey-Fuller test statistic	1% critical value	5% critical value	Test result
Content exploration	0.36	-3.47	-2.87	Non-stationary
First-differenced content exploration	-7.83	-2.57	-1.94	Stationary
Degree centrality	-2.78	-4.01	-3.43	Non-stationary
First-differenced degree centrality	-4.39	-3.47	-2.87	Stationary
Closeness centrality	-1.86	-4.01	-3.43	Non-stationary
First-differenced closeness centrality	-11.42	-3.47	-2.87	Stationary
PageRank	-1.81	-4.01	-3.43	Non-stationary
First-differenced PageRank	-12.50	-3.47	-2.87	Stationary
Content length	-2.62	-4.01	-3.43	Non-stationary
First-differenced content length	-12.40	-3.47	-2.87	Stationary
Constraint	-3.09	-4.01	-3.43	Non-stationary
First-differenced constraint	-4.35	-3.47	-2.87	Stationary
Effective size	-2.67	-4.01	-3.43	Non-stationary
First-differenced effective size	15.40	-3.47	-2.87	Stationary
Density	-1.73	-4.01	-3.43	Non-stationary
First-differenced transitivity	-12.28	-3.47	-2.87	Stationary

For brevity, this table only presents variables included in the final model.

### 3.4 Results

Descriptive statistics for the measured constructs are reported in Table 4.

**Table 4:** Descriptive statistics and correlations.

	Mean	St. Dev.	1	2	3	4	5	6	7	8	9	10	11	12	13	14	15	16	17	18	19	20	21
1. Content length	4,810.19	79.51	1	0.99	0.99	0.96	0.84	0.87	0.97	-0.72	0.3	1	-0.25	0.76	-0.52	-0.06	0.36	-0.26	-0.11	-0.43	-0.25	-0.44	-0.78
2. Num of references	7.58	0.36		1	0.99	0.98	0.86	0.88	0.96	-0.69	0.27	0.99	-0.23	0.77	-0.51	-0.03	0.36	-0.23	-0.14	-0.44	-0.25	-0.46	-0.74
3. Num of internal links	40.41	0.95			1	0.97	0.84	0.88	0.96	-0.72	0.3	0.99	-0.26	0.78	-0.53	-0.05	0.39	-0.26	-0.11	-0.46	-0.28	-0.48	-0.76
4. Num of templates	12.12	0.56				1	0.89	0.89	0.92	-0.59	0.17	0.95	-0.2	0.78	-0.45	0.02	0.37	-0.18	-0.19	-0.45	-0.28	-0.48	-0.64
5. Num of categories	4.29	0.1					1	0.94	0.81	-0.35	-0.09	0.83	0.002	0.55	-0.19	0.21	0.11	0.03	-0.36	-0.24	-0.06	-0.27	-0.41
6. Images by length	0.000419	0.0001						1	0.86	-0.49	0.03	0.87	-0.09	0.59	-0.31	0.11	0.19	-0.08	-0.27	-0.29	-0.12	-0.31	-0.56
7. Num of lvl2 headings	7	0.09							1	-0.77	0.39	0.98	-0.18	0.77	-0.47	-0.11	0.24	-0.25	-0.14	-0.28	-0.13	-0.29	-0.83
8. Flesch reading score	47.3	0.17								1	-0.8	-0.74	0.35	-0.66	0.54	0.37	-0.34	0.46	-0.19	0.28	0.19	0.25	0.92
9. Coleman Liau index	15.1	0.03									1	0.32	-0.4	0.52	-0.42	-0.58	0.34	-0.55	0.43	-0.17	-0.18	-0.15	-0.67
10. Difficult words	231.37	3.44										1	-0.24	0.75	-0.51	-0.08	0.34	-0.26	-0.11	-0.4	-0.22	-0.41	-0.79
11. Degree centrality	0.21	0.09											1	-0.51	0.38	0.76	-0.89	0.93	-0.88	0.82	0.78	0.8	0.28
12. Closeness centrality	0.002	0.001												1	-0.54	-0.37	0.65	-0.52	0.28	-0.57	-0.52	-0.59	-0.67
13. Betweenness centrality	0.001	0.001													1	0.12	-0.53	0.3	-0.21	0.57	0.65	0.56	0.57
14. Eigenvector centrality	0.36	0.08														1	-0.5	0.92	-0.82	0.33	0.36	0.32	0.38
15. PageRank	0.04	0.01															1	-0.74	0.72	-0.94	-0.9	-0.93	-0.25
16. Hub	0.29	0.09																1	-0.85	0.64	0.6	0.63	0.43
17. Constraint	0.36	0.05																	1	-0.56	-0.63	-0.52	-0.1
18. Transitivity	0.55	0.11																		1	0.9	0.99	0.2
19. Effective size	0.8	0.16																			1	0.9	0.14
20. Density	0.66	0.08																				1	0.19
21. Content exploration	11.45	5.62																					1

The estimates for the baseline ARIMA model and transfer function models that considers exogenous variables are reported in Table 5. Note that the ARIMA model and transfer function models are two distinct techniques, and coefficients generated in Model 1 (using ARIMA model) and Model 2 and 3 (using transfer function models) should not be directly compared against each other. ARIMA model is a univariate model that only deals with the outcome series, and TF models are regression techniques that use exogenous variable series to predict the outcome series. They were presented side-by-side only for the purpose of comparing how the response series could be modeled beyond simple ARIMA technique.

**Table 5:** Model estimates predicting content exploration.

	Model 1: ARMA model	Model 2: TF model with only dependent series	Model 3: TF model with input series
Intercept	-0.15 (0.004) ***	-0.02 (0.02)	-0.09 (0.02) ***
*Endogenous series*:			
Content exploration ‐ AR (1)	0.93 (0.007) ***		
Content exploration ‐ MA (1)	-0.99 (0.02) ***		
Content exploration ‐ lag 1		0.31 (0.02) ***	0.23 (0.07) ***
Content exploration ‐ lag 2		0.12 (0.08)	0.12 (0.07) *
Content exploration ‐ lag 3		0.18 (0.07) **	0.16 (0.06) **
*Content-based exogenous series*:			
Content length			0.01 (0.005) ***
Content length ‐ lag 2			0.008 (0.005) ***
Content length ‐ lag 10			0.01 (0.005) ***
*Network-based exogenous series*:			
*H1 Network embeddedness*			
Degree ‐ lag 9			1.92 (0.72) ***
Closeness ‐ lag 7			724.56 (174.06) ***
PageRank–lag 2			84.13 (28.76) ***
*H2 Network connectivity*			
Density–lag 2			5.90 (2.01) ***
Density–lag 6			-2.51 (1.20) **
*H3 Network redundancy*			
Constraint–lag 2			-4.35 (2.10) **
Effective size–lag 7			1.30 (0.42) ***
Adjusted *R*^*2*^	0.37	0.28	0.41
*RMSE*	4.64	4.50	3.38
*AIC*	0.88	0.82	0.50
*BIC*	0.93	0.90	0.78
Log likelihood	-68.36	-61.93	-23.32
F-statistic	48.94 ***	20.14	6.02 ***

*p <0.1

**p < 0.05

***p < 0.01. Standard errors in the parentheses.

Model 2 shows a TF model with only past values of content exploration as predictors to explain the current value of content exploration. Content exploration is a significant predictor of itself at lag 1 (0.31, *p* < .01), and lag 3 (0.18, *p* < .05). It shows that the past history of content exploration matters for its current value.

A final model is returned by automatically selecting an optimal combination of lag lengths for each of the significant predictor variable, judging by the information criterion of *AIC* values. The best model obtained is presented as Model 3 of Table 5. There are seven significant exogenous variables left in the final model.

H1 stated that over time, higher levels of network embeddedness in a knowledge creation system will cause subsequently higher levels of content exploration. This hypothesis received support. There are three metrics describing network embeddedness that are positive and significant predictors of content exploration levels in the future. Degree centrality is a positive and significant (1.92, *p* < .01) predictor of content exploration at lag 7. Closeness centrality is a positive and significant (724.56, *p* < .01) predictor of content exploration at lag 7. PageRank centrality is a positive and significant (84.13, *p* < .05) predictor of content exploration at lag 2.

H2 examines whether higher levels of network connectivity affect content exploration in the future. Density positively predicts content exploration at lag 2 (5.90, *p* < .05) and negatively predicts content exploration at lag 6 (-2.51, *p* < .01). In predicting content exploration, the density value at a more recent time point (i.e., two weeks ago) plays a significant role, while the density value at a more remote time point (i.e., six weeks ago) also contributes to the process. Notably, the more recent impact carries greater magnitude, leading to an overall positive net effect of network connectivity on content exploration. In simpler terms, the larger coefficient at lag 2 counteracts the negative coefficient at lag 6. Articles with densely connected neighborhoods are more likely to explore a wide variety of options in the process of content development. This lends support to H2(a).

H3 stated that over time, lower levels of network redundancy in a knowledge creation system will cause subsequently higher levels of content exploration, which is also supported. Constraint is a negative and significant predictor of content exploration at lag 2 (-4.35, *p* < .01). Effective size is found to positively predict content exploration at lag 7 (1.30, *p* < .05). Higher effective size is reflecting rich and diverse (less redundant) network connections, so this also supports the hypothesis. More redundant network connections are harmful for content exploration in the future.

Overall, Model 3 generated the best model in predicting content exploration. The *R*^*2*^ of Model 3 is 0.41, the highest among all three models. In addition, *RMSE* of Model 3 is the smallest (*RMSE* = 3.38). Also, the *AIC* (0.50) and *BIC* (0.78) values of Model 3 are smaller than other models, indicating a good model fit.

Additionally, two tests were conducted to ensure that the final model did generate random residuals. First, a Ljung-Box test (*Q* statistic = 1.46, *p* = 0.23) suggests that these autocorrelations were not significantly different from zero and the residuals are white noise. Second, an ADF test of the residuals (test statistic = -5.14, *p* = 0.01) confirmed the stationarity of residuals. Overall, the model fitness is satisfactory.

To further analyze the relative contribution of each set of exogenous variables, three additional models were presented in Table 6, which shows separate models that consider one type of network-based exogenous variable at a time. Specifically, the model that adds network embeddedness related variables (H1) can explain nine percent more variance compared to the baseline model in Table 6. The model that adds only network connectivity (H2) can explain six percent more variance compared to the baseline model. The model that adds only H3 related variables about network redundancy can explain one percent more variance than the baseline model. Judging by this analysis, network embeddedness is the set of network-based exogenous variable that contributes most to explaining future values of content exploration. (Note that the three separate models in Table 6 should not be interpreted as stepwise models. The regression terms of the three separate models were extracted from the full model just for the purpose of comparisons).

**Table 6:** Examining each network-based endogenous series for predicting content exploration.

	Baseline: with endogenous and content-based series	Model 1: Adding only H3(a) input series	Model 2: Adding only H3(b) input series	Model 3: Adding only H3(c) input series	Full model
Intercept	-0.09 (0.03) **	-0.12 (0.03) ***	-0.11 (0.03) ***	-0.11 (0.03) ***	-0.09 (0.02) ***
*Endogenous series*:					
Content exploration ‐ lag 1	0.24 (0.08) ***	0.16 (0.08) **	0.188 (0.08) **	0.23 (0.08) ***	0.23 (0.07) ***
Content exploration ‐ lag 2	0.08 (0.08)	0.10 (0.07)	0.10 (0.07)	0.10 (0.08)	0.12 (0.07) *
Content exploration ‐ lag 3	0.08 (0.07)	0.12 (0.07)	0.12 (0.07)	0.06 (0.07)	0.16 (0.06) **
*Content-based exogenous series*:					
Content length	0.01 (0.005) *	0.01 (0.005) **	0.01 (0.005) **	0.009 (0.005) *	0.01 (0.005) ***
Content length ‐ lag 2	0.006 (0.005)	0.006 (0.005)	0.006 (0.005)	0.006 (0.005)	0.008 (0.005) ***
Content length ‐ lag 10	0.01 (0.005) **	0.01 (0.005) **	0.01 (0.005) **	0.01 (0.005) **	0.01 (0.005) ***
*Network-based exogenous series*:					
*H1 Network embeddedness*					
Degree ‐ lag 9		1.78 (0.77) **			1.92 (0.72) ***
Closeness ‐ lag 7		592.79 (174.98) ***			724.56 (174.06) ***
PageRank–lag 2		1.06 (11.70)			84.13 (28.76) ***
*H2 Network connectivity*					
Density–lag 2			1.93 (1.26)		5.90 (2.01) ***
Density–lag 6			-3.9 (1.28) ***		-2.51 (1.20) **
*H3 Network redundancy*					
Constraint–lag 2				-0.24 (1.20)	-4.35 (2.10) **
Effective size–lag 7				0.54 (0.24) *	1.30 (0.42) ***
Adjusted *R*^*2*^	0.24	0.33	0.30	0.25	0.41
Δ*R*^2^ (compared with baseline)		0.09	0.06	0.01	0.17
*RMSE*	4.05	3.82	3.91	4.03	3.38
*AIC*	0.70	0.63	0.66	0.68	0.50
*BIC*	0.84	0.83	0.84	0.84	0.78
Log likelihood	-46.68	-37.91	-41.46	-42.84	-23.32
F-statistic	4.04***	4.89***	4.47***	3.22***	6.02 ***

*p <0.1

**p < 0.05

***p < 0.01. Standard errors in the parentheses.

## 4. Discussion

Knowledge collaboration networks have a set of new characteristics that challenge our understanding of how knowledge evolves. Guided by cultural evolution theory, this article provides a novel and systematic examination to knowledge evolution in open collaboration systems, especially whether and why network-based characteristics drive knowledge evolution. It provides two different ways of understanding network-driven knowledge evolution–evolution pressure examined at the population level (study one), and content evolution trajectory at the artifact level (study two). This article shows that network configurations matter for understanding knowledge evolution, not just as a metaphor, but also as concrete empirical conclusions.

The current study provides several important theoretical implications to the stream of literature on cultural evolution.

### 4.1 Population-level knowledge evolution: Not necessarily network-driven

Study one is a replication of a recent theoretical development in organizational evolutionary theory [7], which suggested that network-based characteristics are more important in driving evolutionary outcomes than (traditional) content-based characteristics. The current study did not support this hypothesis, as the result shows no significant difference between content-based and network-based characteristics in driving evolutionary outcomes. The network-based characteristics are just as important as the content-based ones, which emphasized the importance of considering both types of characteristics when analyzing content evolutionary processes. Further, this study examined a total of 10 network configurations along three theoretical dimensions–network embeddedness, network connectivity, and network redundancy. The results show that overall, different network configurations influence the evolutionary outcomes at different rates. Network connectivity identifies the strongest selection pressure compared to the other two types of network configurations (network embeddedness and network redundancy).

This study was inspired by a recent development in the realm of socio-cultural evolution that there are analytical gains when taking into account how the network traits as a driving for of evolutionary changes [7]. Though the current research found no evidence to support the previously established theory that network-driven evolution is stronger than content-driven evolution, it does not mean that network-driven evolution is not important; instead, based on what can be found in this study, network-driven evolution is just as important as content-driven evolution. It is not surprising because, for knowledge development, the content still holds high priority and network traits also serves critical roles by facilitating contributors’ information flow and exchange. Neither type of traits seems to be the sole leading force in knowledge evolution.

### 4.2 Artifact-level knowledge evolution: Explained by content exploration trajectory

Study two empirically showed how network configurations direct influence knowledge content development, where the evolution trajectory was conceptualized as the process of searching and exploring a wide solution space to develop high quality knowledge product. Time series models showed that network configurations have significant predictive value for content exploration levels in the future. Network configurations are precedents of contributors’ collective strategy of knowledge development. Understanding the structures of collaborators’ networks will directly impact the content development trajectory in the future.

Specifically, network embeddedness (represented by degree centrality, closeness centrality, and PageRank centrality) facilitates the flow of information via editors working on different projects and ultimately helps with the combination and exchange of information. The result adds support to the general notion that diversity of information leads to higher exploration.

Network connectivity, measured by density, is also found to have positive influence on the level of content exploration. For articles that have a well-connected local neighborhood, they are more likely to have access to diverse and useful information from its neighboring articles. The rich experiences of editors in a topic area, manifested as a well-connected local neighborhood, can help the article to explore more content options.

Network redundancy’s negative influence on content exploration received support. Two indicators of network redundancy, constraint, and effective size, was found to negatively predict content exploration, as expected. High network redundancy means that the network contains much repeated information and they are not as useful in facilitating higher levels of content exploration. This again confirmed a general belief that diversity of network connection is helpful for increasing exploration levels, while repetitive information may harm a network’s ability to explore.

This study translates the “evolution of knowledge” from an abstract metaphor into a concrete measurement of knowledge development trajectory and established the dynamic relationship between network configurations and artifact evolution trajectory. By adopting a novel way to quantify how a working group pushes the artifact in a solution space while searching for an ideal output, this study showed that how editors collaborate, and their interaction activity patterns will directly leave traces in the development path of their collective output. This finding offers a direct explanation as to why network configurations matter for informational product development–due to its impact on the level of content exploration realized in a space made up of all potential solutions.

### 4.3 Adding nuances to social network metrics interpretation

The current study also adds nuances to the social network literature by providing a comparison about which set of network configurations might be relatively more influential in driving evolutionary change. While there are many network signals that have been considered important and commonly used in prior research about Wikipedia networks or co-creation networks in general [93], most of these studies examine a few network signals in isolation. This study compared 10 commonly used network metrics in their relative influences in driving evolutionary change. The results showed that network connectivity identifies the strongest selection pressure, compared to the other two network-based characteristics (network embeddedness and network redundancy). It is somewhat surprising because network embeddedness is a widely-used choice when talking about network structural signals [94] in association with production outcomes of knowledge creation systems. The current study encourages researchers to move beyond our traditional preference for the embeddedness-related metrics and consider a wider range of network metrics, especially when applying network embeddedness measures to understand a non-human network (such as knowledge artefact networks). More thinking is needed about context-specific explanations as to what these network metrics actually represent under different network construction methods.

### 4.4 Methodological strengths

The research design employed in this study has at least three distinct advantages. First, the present research represents a unique approach to the study of evolutionary changes in an online knowledge creation community. The Price equation was introduced as a tool that can directly capture the amount of evolutionary change based on different ways of measuring the population’s characteristics. Second, the longitudinal research design allows for a much-needed examination of the temporal link between network configurations and content exploration. This article provides convincing evidence of time-ordered causality (though not strict causality) because it eliminated the ambiguities in interpreting cross-sectional correlations as causal. The fact that lagged effects exist necessitates future exploration of how an online community operates in a temporal framework. Third, this study adopts a new way to quantify the level of content exploration using a novel method [51], and for the first time, applied the method in association with network configurations. Researchers have long been keen to analyze the ways that different content creation strategies are adopted by different knowledge creation communities or solution-seeking activities [42, 86, 87]. This text-based measurement of content exploration was one of the new developments in this direction.

### 4.5 Limitations and future work

This study has several limitations that are worth noting. First, it used only one WikiProject dataset and only considered one type of network among the articles–the editor to article network. Changing the WikiProjects of interest and network construction method may change the empirical findings. These research choices limited the generalizability of the results.

Second, the current study did not exhaust the wide range of network metrics to be included. In the prior investigation of [7], they used nearly 20 different network metrics and this research used only half of them. For this research, however, the purpose was to identify what network metrics matter the most at identifying evolutionary forces and why; thus, the focus was on a much smaller selection of network metrics. Even though justifications for making these selections are provided above based on literature review, it is still unclear what the empirical ramifications are of this selection of network metrics.

Third, this article only showed that network configurations lead to consequential changes in content exploration. Ideally, knowledge quality change should be included in the same model as a dependent variable and content exploration will be tested as the mediator [95]. Then a theoretical model linking network configurations to content development to the performance of content (quality or quantity) can be formally examined. This is an important future direction that can potentially lend more support to the current conclusions.

Fourth, in study one, the fitness measure employed was page views. While this is a commonly used indicator to assess the "success" of online knowledge products [96, 97], it is not without limitations. Cultural evolutionists have utilized more accurate fitness measures, focusing on human cognition outcomes, such as the adoption of information in real-life scenarios [17, 65]. It is important to acknowledge that this article aims to leverage observational data for analyzing real-world cultural evolution. As such, the results obtained should be considered in conjunction with other popular approaches within the cultural evolution field. By recognizing the various methodologies and fitness measures employed in this domain, a comprehensive understanding of the findings can be achieved.

Fifth, our theorization suggests that network configurations might influence the trajectory of content exploration. However, the current empirical design cannot determine the direction of this influence or identify strict causality [98]. It becomes difficult to ascertain whether such network autocorrelation is a direct result of actors’ behavior (in this case, editors’ collaboration activities), or if it stems from the artifacts’ prior characteristics (the level of content exploration), which may attract future editors to engage in the project. A potential future solution involves employing network statistical analysis tools like the SAOM/SIENA approach to better isolate and identify the underlying mechanisms of network temporal changes [99].

## 5. Conclusion

This research can be understood from two broad perspectives: the evolutionary dynamics exhibited in networks, and the networked nature of evolution. The networked nature of evolution is explored in study one, where different kinds of network metrics were examined to identify which ones are particularly important in understanding cultural evolution. The results suggested that network traits are indeed prominent drivers of evolutionary changes, though not more so than content-based traits. The evolutionary dynamics exhibited in communication networks are mostly presented in study two, where time series regression techniques were used to model how temporal changes in network patterns leads to consequential changes in content development trajectories. The collaboration networks were analyzed as having temporal effects on how knowledge evolves over time.

## Supporting information

S1 File Edit dynamics of the WikiProject. (DOCX)

S2 File Operationalization of content exploration. (DOCX)
