# Supplementary material for: Network traits driving knowledge evolution in open collaboration systems
Source: PLoS One. 2023 Nov 14;18(11):e0291097. doi: 10.1371/journal.pone.0291097 (PMC10645342; doi:10.1371/journal.pone.0291097)
Supplement: S1 File — (DOCX) [file pone.0291097.s001.docx]

**S1. Edit Dynamics of the *WikiProject Aquarium Fishes***

Two key observations emerge from a review of the editing/viewing dynamics of this WikiProject.

Firstly, the volume of weekly activities, including pageviews and article revisions, remains relatively stable throughout the analyzed period. This suggests that the overall popularity of the WikiProject did not significantly fluctuate over time. S1 Figure 1 illustrates the distribution of weekly pageviews (log-transformed) across all 394 articles, while S1 Figure 2 shows a log-transformed distribution for weekly total revisions. This transformation reduces variability and prevents data visualization from appearing overly stretched in the graph. The actual analysis results reported in the manuscript did not utilize log-transformed data.

Secondly, the dataset reveals a relatively stable community of editors participating in this project. The dataset encompasses 2610 unique users, consisting of both registered accounts and unique IP addresses. Note that some registered users may choose not to log in, leading to their activities being recorded under an IP address. Moreover, identifying multiple IP addresses belonging to the same user is challenging, as changes in computer network proxy settings can create discrepancies. Among the 2610 users, 1679 (64.3%) are one-time users, while 931 (35.7%) are repeating users who contributed more than once.

The histogram of S1 Figure 3 displays the distribution of revision counts by editor counts. A higher percentage of repeating editors within the WikiProject enhances the meaningfulness of the information flow network constructed by these individuals. In contrast, when the percentage of repeating editors is low, information exchange and the accumulation of experiences become less likely to occur. This situation can arise when most editors are one-time users who visit the WikiProject for random reasons (e.g., from a Google search) and may not return in the future.


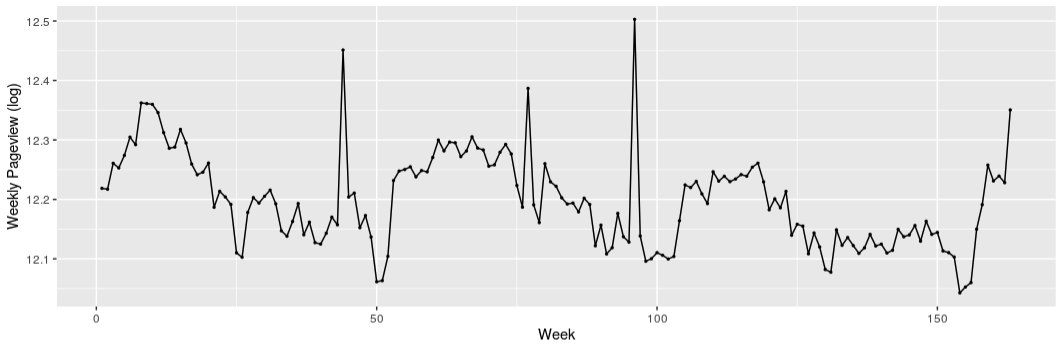


S1 Figure 1. Total Pageviews per Week. The X-axis represents the sequence of weeks from Week 1 to Week 163). The Y-axis value is the weekly total pageviews (natural log transformed).


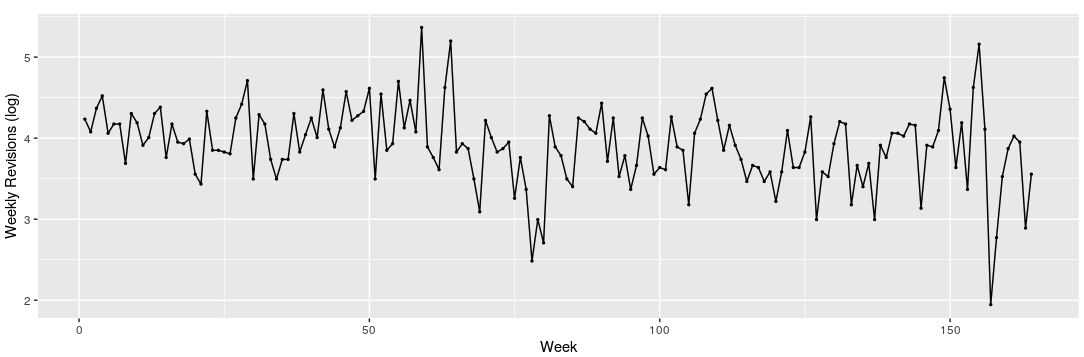


S1 Figure 2. Total Revisions per Week. The X-axis represents the ID of the week (from Week 1 to Week 163). The Y-axis value is the weekly total revisions (natural log transformed).


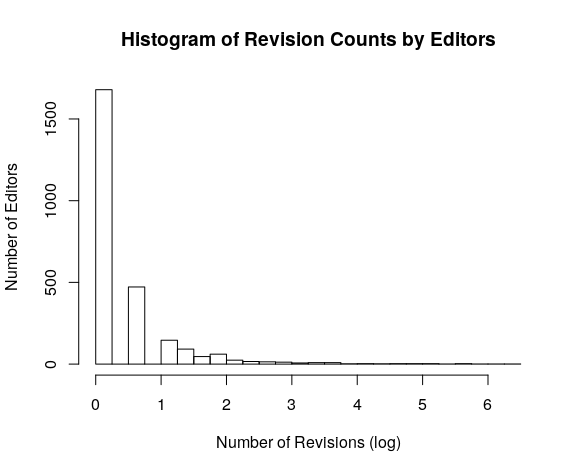


S1 Figure 3. Histogram of Revision Counts by Editors. X-axis shows the number of revisions (natural log transformed) made by an editor. Y-axis shows the count of editors corresponding to each level of “number of revisions (log)”.
