# Supplementary material for: Network traits driving knowledge evolution in open collaboration systems
Source: PLoS One. 2023 Nov 14;18(11):e0291097. doi: 10.1371/journal.pone.0291097 (PMC10645342; doi:10.1371/journal.pone.0291097)
Supplement: S2 File — (DOCX) [file pone.0291097.s002.docx]

**S2. Operationalization of Content Exploration**

The operationalization of content exploration follows the approach introduced in Arazy et al. (2020). Though their study provided a detailed explanation of this construct, to introduce this relatively new concept to a broader research community, this appendix still uses a made-up development trajectory (in S2 Figure 1) to illustrate the operationalization of this construct and its companion R code realization. The notations used here follows that used in Arazy et al. (2020) for consistency.

Specifically, the data processing includes the following steps.

1. Several versions of the textual artifact (in this made-up example, 11 different versions) were collected. So that the initial blank status (*s_0_*) and the final status (*s_r_*) of data observation period was also determined.
2. Standard linguistic pro-processing was conducted for each version: removing stop words, punctuations, emojis, converting uppercase into lowercase, and stemming.
3. Using the bag-of-words approach, each version (*v_i,_* for *i* = 0, …, r) was represented as a binary vector of length N indicating presence (1) or absence (0) of each word, where N is the size of the complete dictionary.
4. The N-dimension vectors representing each version (*v_i_*) is projected into a two-dimensional vector *s_i_* = (*x_i_, y_i_*), where *x_i_* is the Hamming distance between the current version *v_i_* and the original version *v_0_,* *y_i_* is the Hamming distance between the current version *v_i_* and the final version *v_r_.* Hamming distance is a commonly used way of calculating the distance between two binary vectors, which is mathematically the number of different terms between the two vectors. Since the initial status (*s_0_*) is a blank version, each *x_i_* would correspond to the number of terms in *v_i_,* meaning all terms in *x_i_* did not show up in the initial blank version*.* Also, if the last observation’s has term length of *l,* then *s_r_* = (*l, 0*) as it has *l* different terms compared to the initial status. The initial observation would be *s_0_* = (*0, l*) as it has *l* different terms compared to the last observation status. Put it another way, *x_i_* value represents the distance between the initial version and the current version while *y_i_* value shows the distance between the current version and the final observation.
5. As such, each version can now be represented as a vector *s_i_* = (*x_i_, y_i_*) in a two-dimensional space. These positions collectively represent the development trajectory of an artifact.
6. Content exploration of a particular version equals the distance between the node *s_i_* of coordinate (*x_i_, y_i_*) and the line connected by *s_0_* and *s_r_.* Distances were shown as dashed perpendicular lines from each node to the line connected by *s_0_* and *s_r_.*When the node is located on the line, the distance equals zero, meaning zero deviation from the baseline trajectory connecting the shortest distance between the initial version and the last version.

Here is a made-up trajectory of 11 versions of an artifact. The texts used here were inspired by Arazy et al. (2020) example with slight variations.

1. (blank)
2. Smoking is the main cause of cancer.
3. Smoking is the main cause of cancer. People who smoke die early.
4. Smoking is the main cause of cancer.
5. Smoking is the main cause of cancer. People who smoke are not healthy and die early.
6. Smoking is the main cause of cancer.
7. Smoking is the main cause of all cancers. Studies show a correlation between smoking and lung cancer.
8. Studies indicate that there is a relationship between smoking cigarettes and lung cancer.
9. Smoking is the main cause of all cancers. Studies show a correlation between smoking and lung cancer.
10. Studies indicate that there is a significant relationship between smoking cigarettes and lung cancer.
11. Studies indicate that there is a significant relationship between smoking cigarettes from a young age and lung cancer.

The corresponding *s_i_* nodes were drawn in S2 Figure 1.


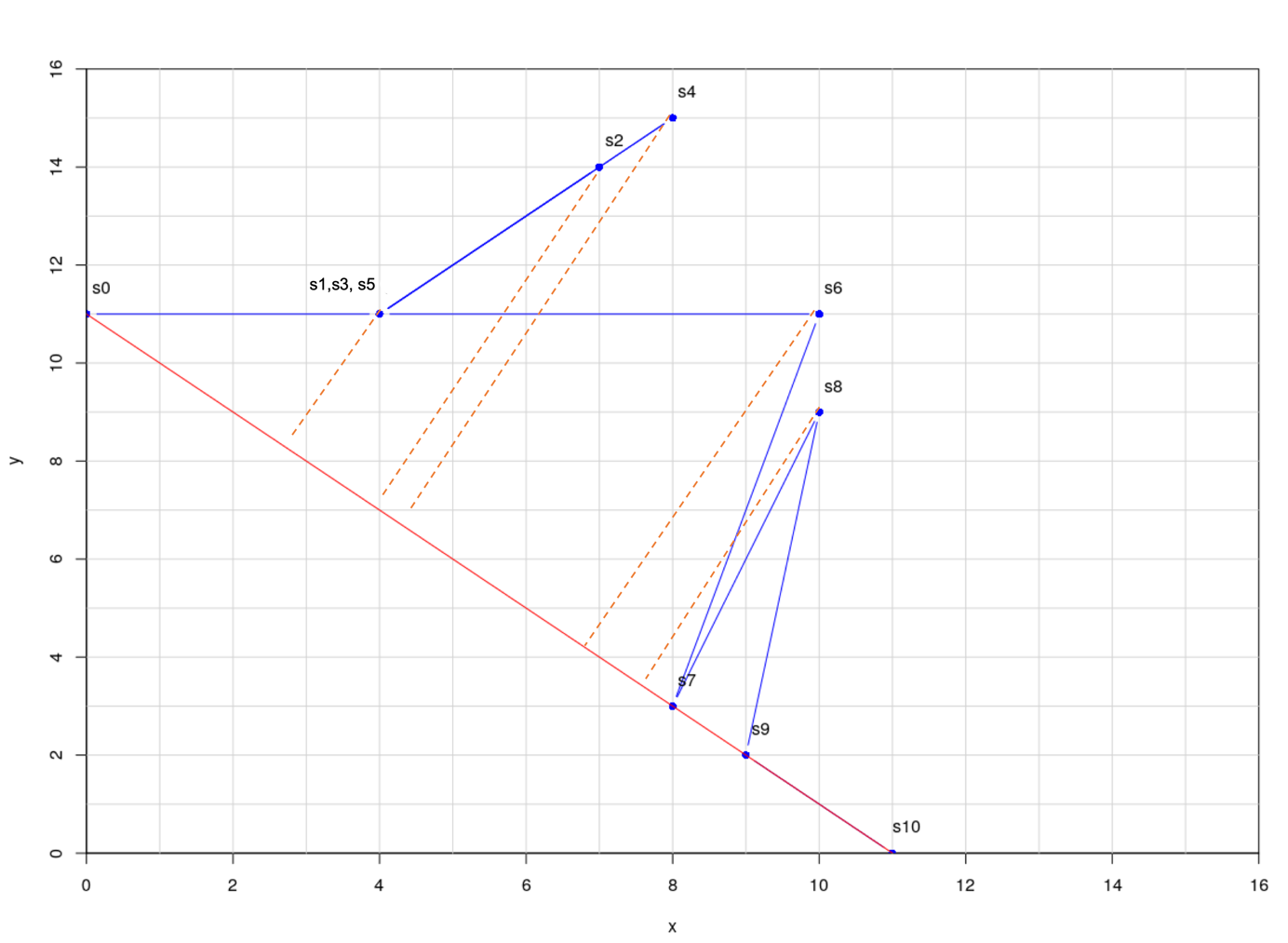


S2 Figure 1. Visualization of a Development Trajectory and Content Exploration. Each version is represented as a point *s_i_* in the graph. Nodes that are further away from the baseline have higher values of content exploration and nodes closer to the baseline have lower exploration value. Those located on the baseline has exploration value of zero.

The specific X and Y coordinates representing each node and the corresponding content exploration value are given in S2 Table 1. The companion R code for the calculations can be found at: https://github.com/RuqinRen/Wiki_Evolution_Exploration/blob/master/appendix_code_companion.R

S2 Table 1. Example Data Point Coordinates and the Calculation of Exploration.

| Status | X | Y | Exploration (distance) |
| --- | --- | --- | --- |
| s0 (blank) | 0 | 11 | 0.000000 |
| s1 | 4 | 11 | 2.828427 |
| s2 | 7 | 14 | 7.071068 |
| s3 | 4 | 11 | 2.828427 |
| s4 | 8 | 15 | 8.485281 |
| s5 | 4 | 11 | 2.828427 |
| s6 | 10 | 11 | 7.071068 |
| s7 | 8 | 3 | 0.000000 |
| s8 | 10 | 9 | 5.656854 |
| s9 | 9 | 2 | 0.000000 |
| s10 (Final) | 11 | 0 | 0.000000 |
